# Study on the Risk Factors of Preoperative Deep Vein Thrombosis (DVT) in Patients With Lower Extremity Fracture

**DOI:** 10.1177/10760296211002900

**Published:** 2021-03-23

**Authors:** Wenjie Chang, Bin Wang, Qiwei Li, Yongkui Zhang, Wenpeng Xie

**Affiliations:** 1 74738 Shandong University of Traditional Chinese Medicine, Jinan, China; 2 Laizhou Hospital of Traditional Chinese Medicine, Laizhou, China; 3 Department of Orthopedic Surgery, 159393 Affiliated Hospital of Shandong University of Traditional Chinese Medicine, Lixia District, Jinan City, Shandong Province, China

**Keywords:** fracture, deep vein thrombosis, DVT, risk factors

## Abstract

**Objective:**

The objective of this work is to discuss and analyze the related factors of lower extremity fracture complicated by preoperative deep vein thrombosis (DVT).

**Methods:**

A total of 11,891 patients with closed fractures of lower extremities were selected. By analyzing each patient’s gender, age, presence or absence of diabetes and hypertension, preoperative plasma D-dimer level, and color Doppler ultrasound of the lower extremity vein, the pertinent factors of the patients with lower extremity fractures complicated by preoperative DVT were analyzed.

**Results:**

A total of 578 with preoperative DVT were detected, displaying a total incidence of 4.86%. All patients were categorized into either the DVT group or non-DVT group. The results demonstrate that there were statistically significant differences between the 2 groups in age, the presence of diabetes and hypertension, the fracture site, and the preoperative plasma D-dimer level (*P* < 0.05). Logistic multivariate analysis revealed that age, the presence of diabetes, and the preoperative plasma D-dimer level of patients were independent risk factors for lower extremity fracture complicated by DVT.

**Conclusion:**

Age, the presence of diabetes, the fracture site, and increased D-dimer levels were found to be potential risk factors and indicators for preoperative DVT in patients with lower extremity fractures. In addition, the preoperative plasma D-dimer level has certain guiding significance for the prediction of venous thrombosis after lower extremity fracture, which is conducive to the early prediction and diagnosis of DVT, but it often must be followed with good clinic acumen and examinations.

## Introduction

Deep vein thrombosis (DVT) is a common complication after trauma, especially after lower extremity fractures.^1^ The incidence of preoperative DVT has been reported to be as high as 32%.^2^ Underlying fatal effects, such as pulmonary embolism (PE), can be caused by DVT, and research has shown that patients treated for venous thromboembolism (VTE) have approximately twice the length of stay and the total cost of hospitalization as those not treated for VTE.^3^ Past studies have shown that DVT in fracture patients is associated with many factors, such as age, sex, the fracture site, and the D-dimer level.^4–6^ For instance, a previous study has shown that DVT is most common in middle-aged patients with lower extremity fractures, but there is no difference in its incidence between sexes.^4^ Different from the results of that study, Williams et al suggested that older age is a risk factor for DVT.^7^ In a study of 462 patients, 71.2% of patients with DVT had hip fractures, 11.9% had tibiofibular fractures, and 10.4% had femoral shaft fractures.^8^ In another study involving 829 patients, the incidences of DVT in patients with hip fractures were found to be only 16.7%.^9^ Therefore, it is necessary to conduct more research to clarify the controversial conclusions of these studies and expand the sample size. The further study on the factors that affect the formation of preoperative DVT in patients with lower extremity fractures is conducive to the formulation of a more reasonable diagnosis and treatment plan, which will allow orthopedic surgeons to choose the optimal operating time.

The purpose of this study was to evaluate the risk factors associated with DVT in patients with lower extremity fractures in the Affiliated Hospital of Shandong University of Traditional Chinese Medicine. Specifically, the potential diagnostic and preventive values of sex, age, fracture sites, chronic diseases, and D-dimer levels in patients with lower extremity fractures were explored via retrospective cohort studies.

## Materials and Methods

### Design, Sample, and Criteria for Participation

In this study, 11,891 patients with lower extremity fractures treated in the orthopedic department of the Affiliated Hospital of Shandong University of Traditional Chinese Medicine from July 2014 to November 2018 were retrospectively reviewed. The inclusion criteria for patients included the following: adult patients with lower extremity fractures (excluding pelvic fractures); patients with closed fractures (non-emergency surgery); fresh fracture patients (hospitalized within 1 week after injury); patients with single fracture. The following patients were excluded: patients under the age of 18; patients with open fractures of the lower extremities; patients with old lower extremity fractures; patients with multiple traumas; patients with a previous history of DVT; patients who required chronic dialysis, therapeutic anticoagulation for any reason, or patients with malignant tumor.

### Data Collection and DVT Diagnosis

Data on the sex, age, previous history of hypertension or diabetes, the preoperative plasma D-dimer level, and the lower extremity fracture site of patients were collected. In this study, fractures were categorized according to the site of trauma into peri-hip fractures (including femoral neck fractures and intertrochanteric fractures, the proximal femur fractures), femoral shaft fractures, peri-knee fractures (including distal femoral fractures, femoral condyle fractures, patellar fractures, and tibial plateau fractures), tibiofibular fractures, and ankle fractures (including ankle joint fractures and foot fractures). The normal range of the preoperative D-dimer level was set at 0-0.5 μg/ml.

All patients were given low molecular weight heparin (LMWH) subcutaneous injection during hospitalization. LMWH was stopped at least 12 h before surgery. Mechanical methods were applied for patients with risk of bleeding before surgery.

According to the guidelines for the prevention of venous thromboembolism in Chinese orthopaedic surgery, Color Doppler ultrasound examination has gradually superseded venography as the primary diagnostic procedure, which is a preferred method for the diagnosis of DVT with high sensitivity and accuracy. All patients underwent the preoperative ultrasound examination of venous thrombosis in the fractured lower extremity 1-2 days before surgery. Regardless of the size of the thrombosis and whether all the vessels were blocked, all patients were treated according to a venous thrombosis diagnosis in the lower extremity, and the location of the thrombosis was recorded. Color Doppler ultrasound was performed in all patients by 1 physician and then reviewed by another physician. Patients with venous thrombosis were classified as the DVT group, while the rest were classified as the non-DVT group.

### Statistical Analysis

SPSS 25.0 software was used for statistical analysis. The incidence rate was expressed as a percentage, independent sample t-tests and χ2 tests were used for statistical analysis, and *P* < 0.05 was considered to indicate a statistically significant difference. If *P* < 0.05 for a factor in the univariate analysis, relevant factors were selected for logistic multivariate regression analysis to determine the factors that affect the occurrence of lower extremity venous thrombosis.

## Results

### Patient Characteristics

A total of 11,891 patients meeting the inclusion criteria were selected and a total of 578 patients had preoperative DVT. These 578 patients were classified as the DVT group, while the remaining 11,313 patients were classified as the non-DVT group. The total incidence of lower extremity venous thrombosis was 4.86%. The patient characteristics are reported in Table 1. No diagnosis related to PE was found in the medical records of all patients during their hospitalization, and no death was recorded.

**Table 1.** Risk Factors for DVT.

Factors	Patients	DVT patients (%)	χ^2^	*P*
Sex			0.113	0.737
Male	5330	263 (4.93)		
Female	6561	315 (4.80)		
Age			49.624	<0.001^a^
18-40	2024	49 (2.42)		
41-60	3480	143 (4.11)		
>60	6387	386 (6.04)		
Diabetes			6.203	0.013^a^
Yes	2667	154 (5.77)		
No	9224	424 (4.60)		
Hypertension			23.023	<0.001^a^
Yes	3568	225 (6.31)		
No	8323	353 (4.24)		
Fracture sites			162.936	<0.001^a^
Peri-hip	4462	282 (6.32)		
Femoral shaft	754	43 (5.70)		
Peri-knee	1776	154 (8.67)		
Tibiofibular	1961	41 (2.09)		
Ankle	2938	58 (1.97)		
D-dimer			2677.305	0.000^a^
0-0.5	8880	12 (0.14)		
0.5-1	451	30 (6.65)		
1-2	257	72 (28.02)		
2-5	397	131 (33.00)		
>5	339	110 (32.45)		

^a^ *P* < 0.05.

### Univariate Analysis of DVT

There were no significant differences in sex between the 2 groups (*P* > 0.05). Also, the incidence of DVT in patients with diabetes or hypertension (5.77%, 6.31%) was found to be higher than that in patients without diabetes or hypertension (4.60%, 4.24%). The χ2 test indicated that there were significant differences in the rate of DVT between patients with and without diabetes or hypertension (*P* < 0.05).

The subjects ranged in age from 18 to 93, with an average age of 61.4 ± 8.29 years. The patients were divided into 3 groups based on age, among which 2,024 belonged to the young adult group (18-40 years old), 3,480 belonged to the middle-aged group (41-60 years old), and 6,387 belonged to the elderly group (over 60 years old). The numbers of DVT cases in each group were 49, 143, and 386, respectively. The average ages of each group were 34.23 ± 5.14 years, 55.81 ± 3.63 years, and 78.36 ± 7.17 years, respectively, and the respective rates of DVT were 2.42%, 4.11%, and 6.04%. The χ2 test results showed that there were significant differences between the rates of DVT in patients of different age groups (*P* < 0.05), which indicates that age is a factor that influences DVT.

**Figure 1.**
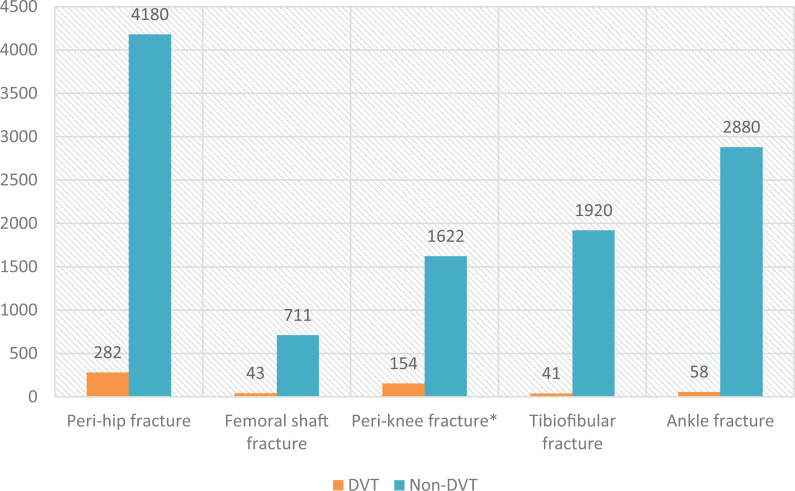
The distribution of DVT in different fracture sites. Peri-knee fracture*: Patients with peri-knee fractures had the highest prevalence at 8.67%.

Of the 11,891 patients, 4,462 had peri-hip fractures, 754 had femoral shaft fractures, 1,776 had peri-knee fractures, 1,961 had tibiofibular fractures, and 2,938 had ankle fractures, and the respective numbers of DVT cases were 282, 43, 154, 41, and 58; thus, the prevalences of DVT were respectively 6.32%, 5.70%, 8.67%, 2.09%, and 1.97%. The χ2 test results showed that there were significant differences in the rate of DVT in patients with different fracture sites (*P* < 0.05), and patients with peri-knee fractures had the highest incidence of DVT (Figure 1).

Among the 11,891 subjects, the plasma D-dimer data of 1,567 patients were excluded due to various reasons, and therefore the number of effective cases was 10,324, among which the number of effective cases in the DVT group was 355. The plasma D-dimer levels of the patients were divided into 5 groups: 0-0.5 μg/ml (8880 cases in the normal group), the 0.5 -1 μg/ml group (451 cases), the 1-2 μg/ml group (257 cases), the 2-5 μg/ml group (397 cases), and the greater than 5 μg/ml group (339 cases). The numbers of patients with DVT in each group were 12, 30, 72, 131, and 110, respectively, and the rates of DVT were 0.14%, 6.65%, 28.02%, 33.00%, and 32.45%, respectively. According to the χ2 test, *P* < 0.05, which indicates that there were significant differences between the rates of DVT in patients with different plasma D-dimer levels.

### Multivariate Analysis of Risk Factors

Multivariate logistic regression analysis further demonstrated the correlations between DVT and age, diabetes, hypertension, the fracture site, and the plasma D-dimer level. Table 2 reveals that age, diabetes, the plasma D-dimer level, and the fracture site were independent risk factors for DVT after lower extremity fractures.

**Table 2.** Multivariate Logistic Regression Analysis Results.

Factors	B value	Standard error	Wald value	*P*
Age	0.453	0.065	48.081	<0.001^a^
Diabetes	-1.754	0.864	4.119	0.042^a^
Hypertension	-0.624	0.755	0.682	0.409
D-dimer	-2.042	0.652	9.810	0.002^a^
Fracture sites	8.761	1.389	39.811	<0.001^a^

^a^ *P <* 0.05.

## Discussion

Virchow’s triad describes the 3 main causes of DVT, namely stasis, hypercoagulability, and endothelial changes.^10,11^ Patients with lower extremity fractures must stay in bed and rest after injury. However, long-term bed rest leads to reduced muscle pumping, severe local blood flow stagnation, and serious vascular endothelial injury. Moreover, the accumulation of coagulation factors leads to the overactivation of the coagulation system, thereby leading to accelerated thrombosis.^8,12–14^

In the present study, patients with lower extremity fractures at the age of >60 years were found to have the highest incidence of DVT (6.04%), and this was similar to the findings of Williams et al^7^ In the opinion of the present authors, there are more internal diseases in elderly patients, which results in a longer preparation time for surgery and longer preoperative resting time. Moreover, elderly patients often have vascular sclerosis, high blood viscosity, and poor venous valve function, which lead to a high incidence of lower extremity DVT.^15^ Li et al found that femoral neck fractures and intertrochanteric fractures are more common in older patients, and that many patients with these fractures choose arthroplasty surgery, which also increases the risk of DVT.^9^

The results of the present study revealed that the prevalence of DVT was significantly higher in the diabetic group than in the non-diabetic group (5.77%:4.60%). Chung et al found that the risk of VTE in diabetic patients is 1.44 times higher than that in non-diabetic patients.^16^ After fracture, the body is in a state of stress, so the blood glucose in the diabetic patients had a large range of changes, thereby leading to the release of a large number of cytokines and further activating the coagulation system in the body, which can also lead to increased platelet activation and damage to the fibrinolytic system.^16,17^ In addition, it is believed that diabetic patients must regulate their blood glucose before surgery, which increases the preoperative preparation time of diabetic patients as compared to that of normal patients.^18^ These factors lead to patients spending more time in bed, thereby limiting preoperative functional exercise and further increasing the risk of DVT. Kang et al^19^ believe that pain, stress reaction, and emotions lead to endocrine abnormalities that accelerate the secretion of epinephrine, endothelin, and 5-hydroxytryptamine, all of which can lead to the increase of hypercoagulability and easily induce DVT.

The results of the χ2 tests conducted in the present study indicate that there were significant differences between the rate of DVT among patients with or without hypertension, but logistic multivariate analysis showed that hypertension was not an independent risk factor in the formation of DVT in patients with lower extremity fractures. It is speculated that this result occurred because older patients accounted for a larger proportion of the subjects, and studies have shown that the prevalence of hypertension increases with age.^20^

Furthermore, in the cohort study, the fracture site was found to be an independent risk factor for DVT in lower extremities after fracture, and the incidence of DVT in patients with peri-hip fractures was 6.32%. Peri-hip fractures are more common in older patients, and many patients with these fractures choose arthroplasty surgery; this is one of the reasons for the high incidence of DVT following peri-hip fractures.^9^ Some researchers have also confirmed that hip fracture is an important risk factor for, and is significantly associated with, the occurrence of DVT.^8^ However, in the present study, patients with peri-knee fractures had the highest prevalence at 8.67%. Peri-knee fractures contain many complicated comminuted fractures caused by violence, which can cause relatively serious vascular endothelial injury in addition to bone injury. Moreover, the fixation of the affected extremity after the injury can impair venous function, and these lesions can lead to a hypercoagulable state.^6^ Thus, patients with peri-knee fractures exhibit the 3 risk factors associated with Virchow’s triad, which is one reason for the high incidence of DVT found in the present study.

When fibrin filaments are degraded by fibrinolytic enzymes during coagulation, plasma D-dimer is produced. Therefore, an increased D-dimer content in plasma indicates the occurrence of thrombosis and dissolution in vivo, and can therefore be used as an indicator of non-invasive thrombosis.^21^ The results of the present study demonstrated that the plasma D-dimer level is an independent factor that affects the formation of DVT, which is similar to the results of Zhang et al^8^ However, the D-dimer level increases with age and elderly patients are more prone to false-positive test results, which reduces the specificity of detection in these patients; therefore, studies have demonstrated the need to set the best cut-off value according to age to increase the specificity of detection.^22^ In addition, any increased fibrin or decomposition process, such as pregnancy, cancer, trauma, inflammation, infection, etc. will increase the D-dimer level; therefore, an increase in the D-dimer level cannot be used for the specific detection of VTE.^23^ The D-dimer level alone is often unreliable for the diagnosis of DVT; however, as a low-cost and readily available biomarker, an elevated D-dimer level is useful for clinicians to identify or exclude VTE in patients based on symptoms and imaging findings.^24^

## Strengths and Limitations of This Study

To our knowledge, this is a retrospective study with the largest sample size on preoperative DVT risk factors in patients with lower limb fractures. Some new conclusions have been drawn, which provide a new reference for clinicians to take measures to prevent DVT. Nevertheless, some limitations still exist. First, the results may be biased by our single-center, retrospective study design. Second, Color Doppler ultrasound examination was used as the diagnostic standard. Although this modality is non-invasive and reproducible, but the gold standard for the diagnosis of DVT is venography, the diagnostic accuracy is inferior to that of venography, which also had a certain effect on the results. Third, age, the presence of diabetes, the fracture site, and increased D-dimer levels were found to be independent risk factors for preoperative DVT in patients with lower extremity fractures, but some other factors, such as body weight index, smoking, heart function insufficiency, lower extremity vein varices, may also affect the occurrence of DVT, while these factors were not studied in the current study.

## Conclusion

In summary, age, the presence of diabetes, the fracture site, and increased D-dimer levels were found to be potential risk factors and indicators for preoperative DVT in patients with lower extremity fractures. In addition, the preoperative plasma D-dimer level has certain guiding significance for the prediction of venous thrombosis after lower extremity fracture, which is conducive to the early prediction and diagnosis of DVT, but it often must be followed with good clinic acumen and examinations. Therefore, it is suggested that orthopedic surgeons analyze whether these risk factors are present in patients with lower extremity fractures to effectively prevent the formation of lower extremity venous thrombosis.
